# Two-stage approach for olfactory groove meningioma

**DOI:** 10.3171/2025.10.FOCVID25185

**Published:** 2026-01-01

**Authors:** Matthieu D. Weber, James Mamaril-Davis, Mark A. Damante, Daniel M. Prevedello

**Affiliations:** 1 The Ohio State College of Medicine, Columbus;; 2 Department of Neurological Surgery, The Ohio State University, Columbus; and; 3 The James Cancer Center, Columbus, Ohio

**Keywords:** olfactory groove meningioma, skull base, endoscopic endonasal approach

## Abstract

A 51-year-old female presented with an olfactory groove meningioma causing seizures, confusion, and anosmia. Large tumor size and frontal lobe edema can complicate resection in a single-stage craniotomy and lead to brain injury secondary to intraoperative retraction. Moreover, complete resection by the endoscopic endonasal approach (EEA) alone is not feasible given the lateral extent of the tumor. A two-stage resection with a transcribriform transplanum EEA, followed by a fronto-temporo-parietal craniotomy 4.5 months later, was performed. The EEA allows for initial decompression, devascularization, and reduction of edema, which may decrease the morbidity of craniotomy by reducing the significance of frontal lobe retraction.

The video can be found here: https://stream.cadmore.media/r10.3171/2025.10.FOCVID25185

## Transcript

This video represents a two-stage approach for olfactory groove meningioma.

### 0:25 Patient Presentation.

We present the case of a 51-year-old female who presented with a 5-year history of seizures, worsening visual acuity, subjective confusion, anosmia, and worsening headaches. Twenty years ago, an MRI from an outside institution was obtained due to concern for optic neuritis and incidentally revealed a 5-mm anterior fossa meningioma. She was lost to follow-up and presented at an outside institution with seizures 15 years later. She presented to us 5 years later with a large anterior fossa lesion showing optic nerve compression. Other than subjectively diminished visual acuity and anosmia, her physical exam was unremarkable.

### 1:00 Background and Rationale.

Olfactory groove meningiomas have historically been resected by unilateral or bifrontal craniotomy.^1^ Expanded endoscopic endonasal approaches offer an alternative access corridor to the anterior cranial fossa and are suitable for certain OGMs.^2^ Transcribriform EEA invariably results in loss of olfaction. A transcribriform EEA should be considered in patients presenting without olfaction and/or patients with extensive frontal lobe edema, particularly with intervening frontal lobe requiring retraction.^3^ However, this approach has anatomical limitations, namely difficult access to and limited mobility in regions of lateral tumor extension, particularly past the orbital apex.^4^ Therefore, in the case of a large OGM with extension lateral to the orbital apex and significant associated edema, we suggest an EEA for initial devascularization and decompression, followed by a unilateral transcranial approach 3–4 months later.^5^ The minimum of a 3-month interval allows for reduction in frontal lobe edema prior to craniotomy. We have observed good outcomes with this staged approach, with improved visual outcomes and potentially improved cognition.^3^

### 2:02 Preoperative Imaging.

Preoperative imaging of this patient showed a large heterogeneously enhancing anterior fossa mass with extension past the orbital apex. There was extensive right frontal lobe edema on the FLAIR sequence. The anterior cerebral arteries were displaced to the left and followed a course posterior to the lesion.

### 2:18 Stage I: Summary of the Endoscopic Endonasal Approach.

For the first-stage transcribriform EEA, the patient was placed in standard three-pin fixation with slight right-sided head rotation and left head tilt, allowing for an ergonomic two-surgeon four-hand technique. We utilized a multidisciplinary approach with our ENT colleagues, who harvested a vascularized nasoseptal flap, performed anterior and posterior ethmoidectomies, frontal sinusotomy, and sphenoidotomy for complete exposure of the planum sphenoidale and cribriform plate. The anterior and posterior ethmoidal arteries were cauterized and divided to devascularize the tumor. Next, we drilled away the hyperostotic bone and performed a wide dural exposure. We then turned our attention to intradural tumor debulking using both ultrasound guidance and neuronavigation. The final steps included a skull base reconstruction using dural substitute, fat graft, and our harvested nasoseptal flap.

### 3:05 Cribriform Dural Exposure.

The fovea ethmoidalis and lateral lamella were carefully drilled off, ensuring preservation of the lamina papyracea. We show the olfactory fimbria being removed from the cribriform plate, after which the latter was drilled to expose the underlying dura. Kerrison rongeurs were then used to enlarge the dural exposure, which is carried anteriorly and laterally. The crista galli is then removed using a combination of drilling and biting with the Kerrison.

### 3:46 Wide Dural Exposure and Hyperostotic Bone Removal.

The dural exposure is extended posterior to the cribriform plate by drilling the planum sphenoidale. This was extended past the opening of the sphenoid sinus all the way to the tuberculum sella. The curvature of the sella and the opticocarotid recesses visible just laterally were used as anatomical landmarks to guide bony removal. We used a drill to get the bone eggshell thin and then a Cottle dissector to fracture the pieces off carefully. Particular attention was paid to the right side, which harbored most of the hyperostotic bone.

### 4:35 Postoperative Extent of Bony Resection on CT Imaging.

Here is the CT representation of the bony removal performed at stage I.

### 4:41 Intradural Debulking.

We then turned our attention to the intradural portion of the procedure. The dura was incised sharply in the midline using a feather blade and the opening expanded with scissors. A combination of suction and side aspiration cutting devices were used to debulk the tumor. We debulked the tumor centrally. We continued this process until we felt the tumor became soft and pliable, indicating proximity to the capsule. The endoscopic ultrasound was used to guide debulking in order to avoid violating the brain-tumor interface and risk damaging the anterior cerebral arteries. After debulking, we found a small CSF leak to the left of the tumor interface, which was repaired using clips. Hemostasis was obtained before working on our reconstruction.

### 5:23 Skull Base Reconstruction.

For the reconstruction, dural substitute was placed over the defect. The bottom of the sphenoid sinus was packed with the harvested abdominal fat to buttress the pedicle of the nasoseptal flap. The flap was then draped over this, and nasal packing was placed in the cavity to hold the construct in place.

### 5:41 Stage I: Postoperative Follow-Up.

This patient had an uncomplicated postoperative course and endorsed subjective visual normalization and improved mentation. Following surgery, she was not able to smell, which was expected and unchanged from her baseline. Pathology revealed a mixed WHO grade I and II meningioma with an estimated Ki-67 of 3%–6%. On her postoperative imaging obtained at 1 month, there was a substantial decrease in edema and reduction in mass effect due to central debulking of the tumor.

### 6:06 Stage II: Summary of the Transcranial Approach.

Stage II was performed approximately 4 months after stage I. The patient was placed in standard three-pin rigid fixation and positioned supine with the head turned 20° to the left and in slight extension. We used a bicoronal-style incision and performed a right-sided fronto-temporo-parietal craniotomy. The procedure was divided into two stages: 1) extradural anterior clinoidectomy for right optic nerve decompression and 2) intradural tumor resection. Lastly, the dura was closed and the skull base reconstructed with titanium mesh and plates.

### 6:37 Drilling of the Sphenoid Wing/Orbital Roof and Clinoidectomy.

After the craniotomy, the anterior fossa dura was peeled and the meningo-orbital band was identified and cut. Hyperostosis along the sphenoid wing was drilled. We then drilled the orbital roof until we reached the anterior clinoid. The optic nerve was unroofed superiorly, and the extradural anterior clinoidectomy was performed. Bleeding from the clinoidal space was controlled with hemostatic agent.

### 7:06 Opening of the Opticocarotid Cistern.

After dural opening and proximal sylvian fissure split, we opened the opticocarotid cistern to release CSF and promote brain relaxation. The frontal lobe is on the left side of the screen and the temporal lobe on the right. Immediately, we could visualize tumors superior to the opticocarotid triangle.

### 7:21 Tumor Debulking and Resection.

We began by mobilizing the mass away from the anterior cranial fossa floor and used an ultrasonic aspirator to start debulking the inferolateral pole of the tumor away from the optic nerve. This process was continued until the tumor capsule relaxed and was more easily manipulated. This allowed us to slowly start circumferentially defining the brain-tumor interface. We found significant pial invasion on the right frontal lobe and vessels were coagulated using bipolar forceps. A combination of ultrasonic aspiration and sharp dissection was used to resect the tumor in a piecemeal fashion. As dissection progressed, the mass became easier to manipulate, which allowed us to tackle the superomedial-most attachments to the falx. These attachments, as well as those along the anterior cranial fossa floor, were coagulated and cut.

### 8:52 Hemostasis and Closure.

Thorough hemostasis was achieved. The dura was closed using interrupted sutures along an imbricated dural substitute with an additional piece of dural substitute placed over the repair. The bone flap was secured in place using titanium mesh and plates. The soft tissues were reapproximated in a standard layered fashion with interrupted Vicryl sutures.

### 9:11 Stage II: Postoperative Follow-Up.

Postoperative imaging at 5 months showed a complete tumor resection. The patient did well and had an uncomplicated postoperative course. She retained normal pituitary function and had an unremarkable neurological exam. She continued to report visual improvement.

## Disclosures

Dr. Prevedello reported consulting for Stryker, Integra, Leica Microsystems, and BK Medical; and receiving royalties from KLS Martin, ACE Medical, and Mizuho.

## Author Contributions

Primary surgeon: Prevedello. Assistant surgeon: Damante. Editing and drafting the video and abstract: Weber, Mamaril-Davis, Damante. Critically revising the work: all authors. Reviewed submitted version of the work: all authors. Approved the final version of the work on behalf of all authors: Prevedello. Supervision: Prevedello.

## Supplemental Information

### Patient Informed Consent

The necessary patient informed consent was obtained in this study.
